# Calculation of the Lumbosacral Segment Volume of the Spinal Cord in Ducks (Anas) Using the Stereological Method

**DOI:** 10.1002/vms3.70289

**Published:** 2025-03-21

**Authors:** Gamze Cakmak, Zafer Soyguder

**Affiliations:** ^1^ Department of Anatomy, Faculty of Veterinary Medicine University of Yuzuncu Yil Van Turkey

**Keywords:** Duck, lumbosacral segment, spinal cord, stereology, volume

## Abstract

In this study, white and grey matter volume densities of the lumbosacral part of spinal cord in ducks (Anatinae) were investigated by a stereological method. Ten healthy ducks weighing 3–4 kg, regardless of gender, were used as material. Anesthetized animals were perfused with 10% buffered formaldehyde. The lumbosacral parts of the ducks were exposed by dissecting the spinal cords. The lumbosacral section was segmented. Tissue samples from each segment were determined. A total of 12 sections were taken from these tissue samples, each 250th section with a thickness of 5 µm on the microtome. These sections were stained with haematoxylin‐eosin. Photos were taken with an X10 objective. By using Cavalieri's Principle, volume density calculations of both the whole tissue and the white and grey matter were performed separately in each segment of the lumbosacral part of the spinal cord in ducks. Shtereom I program was used for calculations. As a result, the white matter and grey matter volume values of all tissue in the duck lumbosacral segments and their volume fractions with each other were determined and evaluated in the light of the literatures. In this study, when all the volume values of the lumbosacral spinal segment in duck were revealed, it was determined that the volume value increased in the range of LS3 and LS8 segments. The increase in the whole volume value in these segment ranges in ducks gave rise to the idea that the enlargement called intumescentia lumbosacralis may be between these segments.

## Graphical abstract

In the statistical evaluation made in terms of whole segment volume values, it was determined that there was a difference between the segments. It is seen that there is a statistical difference in terms of white matter and grey matter volume values. Differences were also detected in GM/LSS and GM/WM values. It was stated that the difference between segments was insignificant in WM/LSS value.

In the statistical evaluation of between animal lumbosacral volume values, the difference was insignificant in terms of whole segment and grey matter volume values, while the difference was significant in terms of white matter, GM/WM, WM/LSS and GM/LSS values.

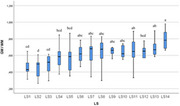

## 1 Introduction

Duck is a species known as *Anas*, which belongs to the Anatidae family of the Anseriformes order of the Aves class. This study revealed a stereological evaluation of the lumbosacral segment of the spinal cord (LSS) in ducks. In poultry, the spinal cord extends along the entire columna vertebralis, starting from the foramen magnum and extending to the coccygeal region, as in mammals (Dursun 2002). However, unlike the mammalian spinal cord, the spinal cord in poultry is included in the so‐called filum terminale without forming the cauda equina formation (Baumel 1975; Dursun 2000; Haziroglu et al. 2001; King and McLelland 1984). In poultry, the spinal cord differs in appearance. While it is in the form of a wide and short organ in mammals, the spinal cord is a long, thin and narrow formation in poultry (Nickel 1977; Dursun 2002). As in mammals, there are membranes called dura mater, arachnoidea and pia mater that surround and support and protect the spinal cord in poultry (Dursun 2002; Junqueira et al. 1998; Russ and Dehoff 1999). It is possible to talk about two expansion zones in poultry. These are known as the intumescentia cervicalis corresponding to the cervical region and the enlargements in the lumbosacral region intumescentia lumbosacralis (Badawi et al. 1994; Haziroglu et al. 2001). While the spinal cord is named cervical, thoracal, lumbosacral and caudal in poultry, the only difference from mammals is that the lumbar and sacral regions are named together (Baumel et al. 1975). White and grey matter (GM) regions are seen in cross‐sections taken from the spinal cord in poultry (Dursun 2002). GM is surrounded by white matter (WM). This changed the appearance of GM to a typical butterfly (Junqueira et al. 1998; Tanyolac 1993). The canal canalis centralis can be seen with the naked eye and is located in the middle of the GM (Dursun 2000; Junqueira et al. 1998). Although stereology is a method used to evaluate biological structures, it is also a science that reveals that objects that are difficult to examine in a three‐dimensional environment can also be examined in two‐dimensional environments (Russ and Dehoff 1999; Sterio 1984; Von Bartheld 2002; Weibel 1981). It can also be defined as a method in terms of stereology and morphometric studies, and neutral methods can be preferred to biased methods (Howard and Reed 1998). Different methods are preferred to calculate the total volumes of organs and structures or the volumes of their components at stereology (Cruz‐Orive and Weibel 1990; Gundersen 1986; Gundersen, Bagger, et al. 1988; Weibel 1981). The Cavalieri's Principle in stereology is widely used in total volume calculations (Gundersen and Jensen 1987; Gundersen, Bendtsen, et al. 1988; Howard and Reed 1998; Canan et al. 2002). There are two types of movements in birds: flying in the air with their front legs, that is, their wings, and walking on the ground with their hind legs. In birds, the ability of birds to walk on two legs on the ground is ensured by a special balance control, as the hind legs are located caudal to the body's centre of gravity and are horizontally oriented (Mittelstaedt 1964; Singer 1884; Trendelenburg 1906; Biederman Thorson and Thorson 1973; Delius and Vollrath 1973). It has been reported in a review that the special glycogen structure in the lumbosacral region can serve as a sensory organ in this way (Grimm et al. 1997; Necker 1999). It was shown that the lumbosacral region serves as a sensory organ in controlling movement and posture while on the ground or moving from the ground in a review. Walking and flying movements are carried out independently of each other. The vestibular system works during flight, and the lumbosacral system works during walking (Necker 2006).

In volume calculation studies, a coefficient of error 10% or less has been reported as a valuable parameter. In this study, whole segment, WM and GM volume values were calculated and the coefficient of error value was found below 0.05 (Garcia‐Finana et al. 2003; Gundersen and Jensen 1987; Gundersen et al. 1999). While it was revealed that the study was reliable in terms of the coefficient of error, it was observed that it was in compatible with the mentioned literature. As a result, it was stated that all volume, white and GM volume densities of the LSS in ducks can be calculated separately and that the obtained values can be revealed by stereological methods.

The main objective of this study was to calculate the whole volume values and GM volume and WM volume of the LSS of the ducks. We also aimed to reveal which segments correspond to lumbosacral enlargement in ducks. Additionally, we aimed to identify the segments corresponding to lumbosacral enlargement, providing a reference for veterinary anatomy, neurobiology and histology.

## 2 Materials and Methods

**FIGURE 1:**
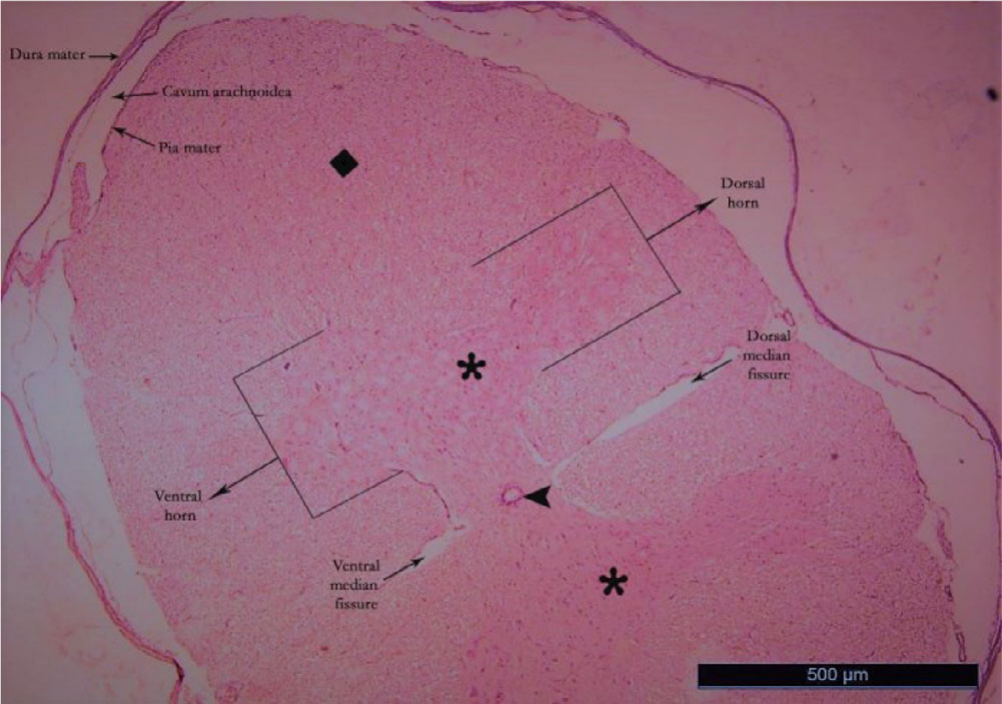
Lumbosacral segment (LS2) of duck (X10) haematoxylin eosin. Square: white matter; star: grey matter; arrowhead: canalis centralis.

**FIGURE 2:**
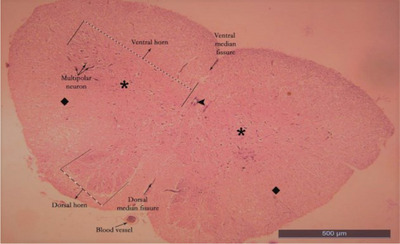
Lumbosacral segment (LS9) of duck (X10) haematoxylin eosin. Square: white matter; star: grey matter; arrowhead: canalis centralis.

**FIGURE 3:**
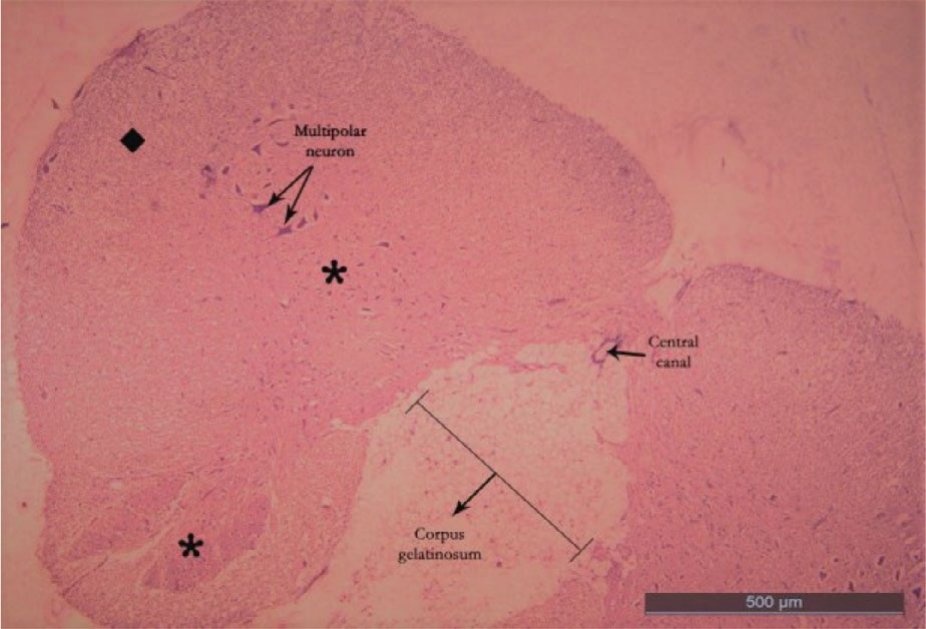
Lumbosacral segment (LS11) of duck (X10) haematoxylin eosin. Square: white matter; star: grey matter; arrowhead: canalis centralis.

**FIGURE 4:**
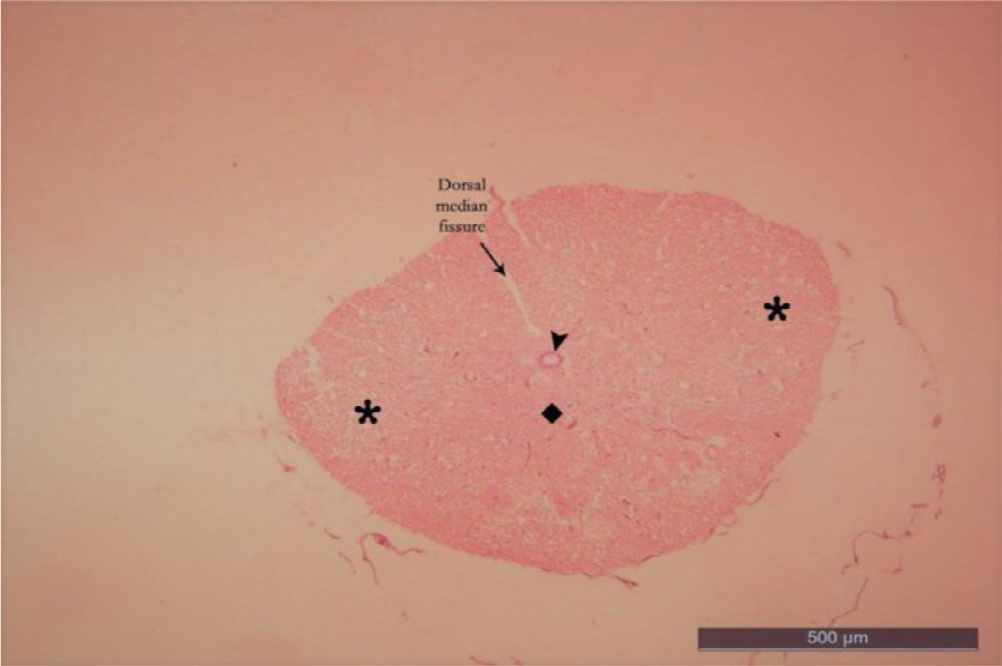
Lumbosacral segment of duck (LS12) (X10) haematoxylin eosin. Square: grey matter; star: white matter; arrowhead: canalis centralis.

Ten healthy ducks with an average weight of 3–4 kg were preferred regardless of gender for this study. Anaesthesia in ducks was achieved with 50 mg/kg ketamine hydrochloride intravenously (Aslanbey et al. 1948). Animals were perfused with 10% buffered formaldehyde from the heart. Ducks were kept in formaldehyde for 1 week to fix the animals. The fixed spinal cords segments were dissected by following the branches of the spinal nerves (Thomas and Comb, 1965). Two important factors of the Cavalieri's Principle were provided for this study. The number of sections and the number of counted points were suitable for this study. Coefficient of variation (CV) and coefficient of error (CE) values obtained in stereological studies were calculated for this study. CV value was under 0.2, and the CE value was under 0.5 (Cruz‐Orive and Weibel 1990; Weibel 1970). It was determined that a total variant square root/total number of points formula was needed in order to be able to sample the sections and calculate the coefficient of error (Gundersen and Jensen 1987). Paraffin‐blocked tissue samples were prepared separately from each of the LSS of the spinal cord removed by dissection, vertically in the same direction. Note that 5‐µm thick sections were taken transversely from the blocked tissues with a Rotary microtome (Leica LM 2135 Nussloch, Germany) by systematic random sampling (SRS) from the beginning to the end of the tissue. While the sections were taken, 12 sections were obtained for each segment of each animal at a stepping ratio of 1/250. The sections were stained with haematoxylin and eosin (Kamfar et al. 2023) (Figures 1, 2, 3, 4; Luna 1968, Ahani and Alizadeh 2024). When the tissue samples were taken, stepping was not considered necessary because the spinal cord of the ducks is a small structure (Canan et al. 2002; Cruz‐Orive and Weibel 1990). First, the sections of the LSS were photographed under X10 objective (Figures 1, 2, 3, 4). Photographed sections were calculated by using the dotted area ruler, first the area and then the volume calculations. The Shtereom I package program was preferred for these calculations (Oguz et al. 2007). Applications of the Cavalieri's Principle were also chosen as a guide for these calculations (Roberts et al. 1998). The shrinkage value of the values obtained as a result of the measurement of the LSS tissue on the preparation with the help of calipers was calculated as 0.32283 with the appropriate formula, according to the average size values kept in formaldehyde. Total volume values, total WM and GM volume values were calculated separately for each segment of 14 LSS present in ducks. For volume value calculations, the number of points was used because the ratio of the points on the cross‐section could be evaluated (Howard and Reed 2005).

### 2.1 Statistical Analysis

In the statistical evaluation made in terms of whole segment volume values, it was determined that there was a difference between the segments (Figures 5, 6, 7, 8, 9, 10, 11, 12, 13, 14, 15, 16). It is seen that there is a statistical difference in terms of WM and GM volume values. Differences were also detected in GM/LSS and GM/WM values. It was stated that the difference between segments were insignificant in WM/LSS value (Table 1).

**FIGURE 5:**
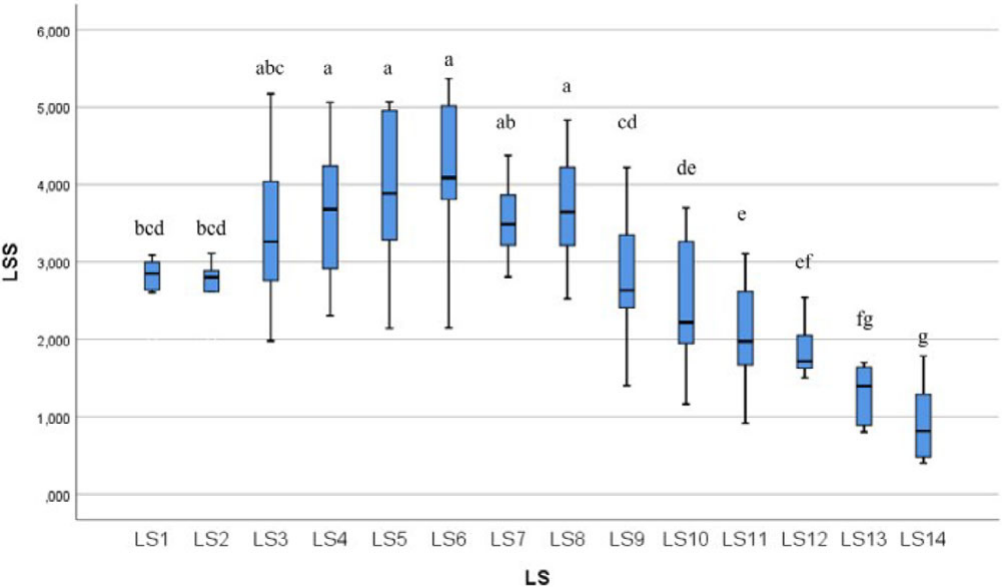
Statistical analysis for LSS, LS.

**FIGURE 6:**
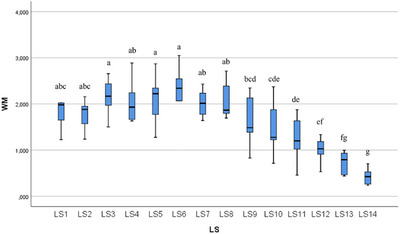
Statistical analysis for WM, LS.

**FIGURE 7:**
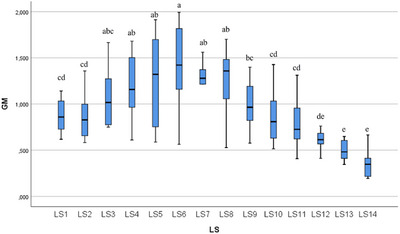
Statistical analysis for GM, LS.

**FIGURE 8:**
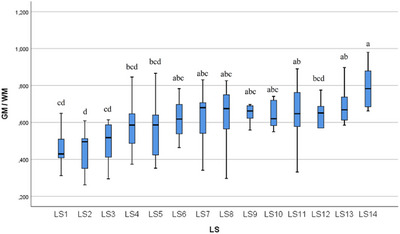
Statistical analysis for GM/WM, LS.

**FIGURE 9:**
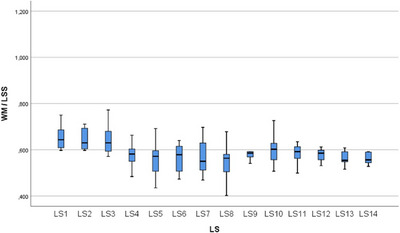
Statistical analysis for WM/LSS, LS.

**FIGURE 10:**
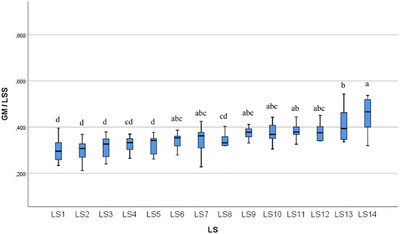
Statistical analysis for GM/LSS, LS. The letters (a, b, c, d) indicates the differences.

**FIGURE 11:**
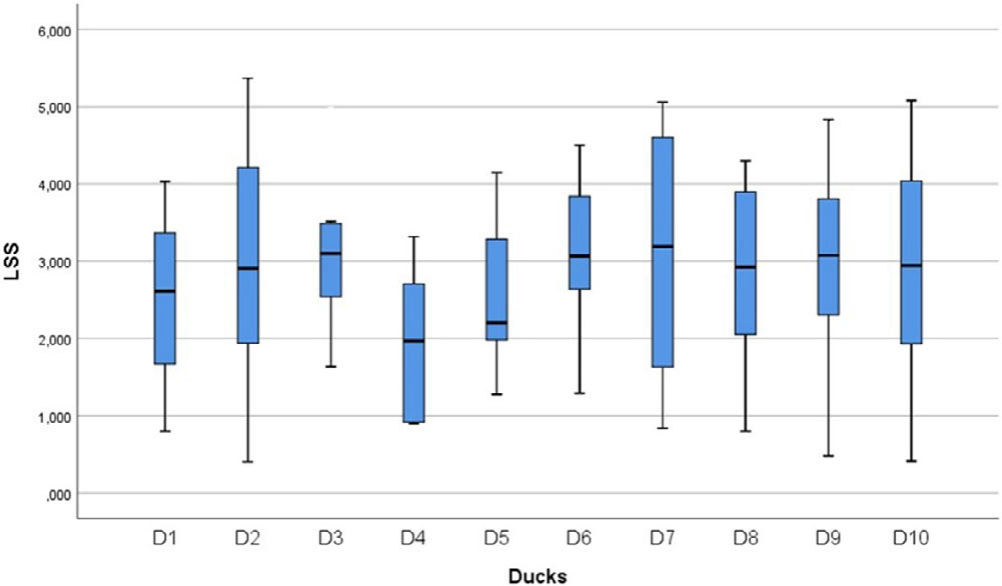
Statistical analysis for LSS, ducks.

**FIGURE 12:**
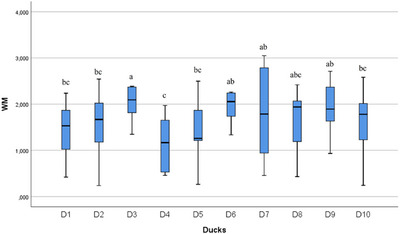
Statistical analysis for WM, ducks. The letters (a, b, c, d) indicates the differences.

**FIGURE 13:**
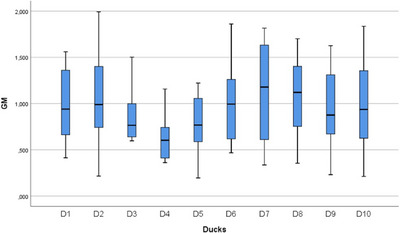
Statistical analysis for GM, ducks.

**FIGURE 14:**
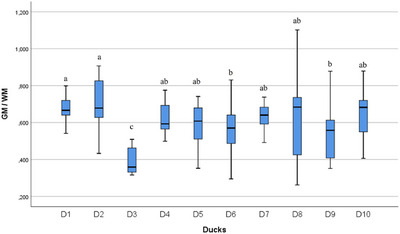
Statistical analysis for GM/WM, ducks. The letters (a, b, c, d) indicates the differences.

**FIGURE 15:**
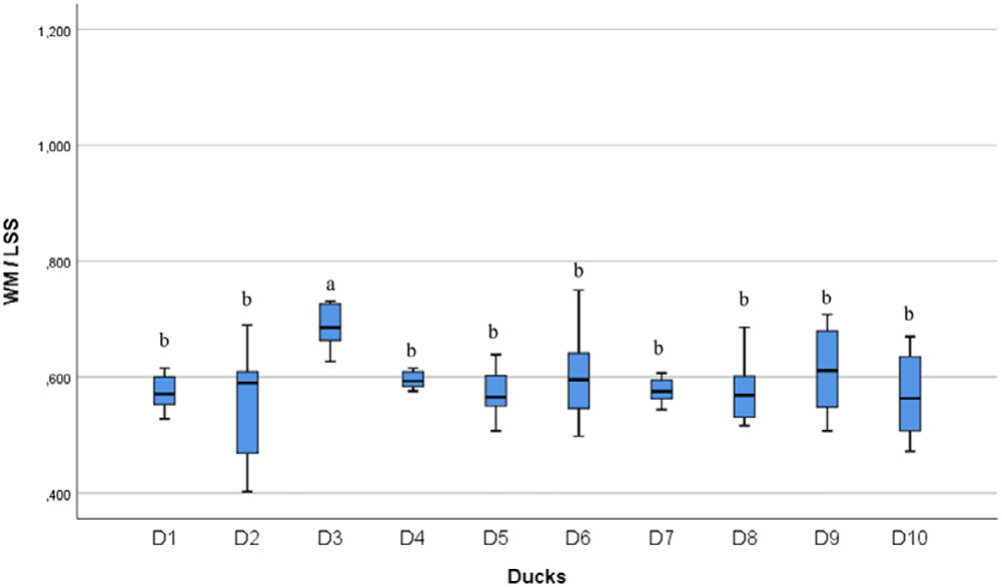
Statistical analysis for WM/LSS, ducks. The letters (a, b, c, d) indicates the differences.

**FIGURE 16:**
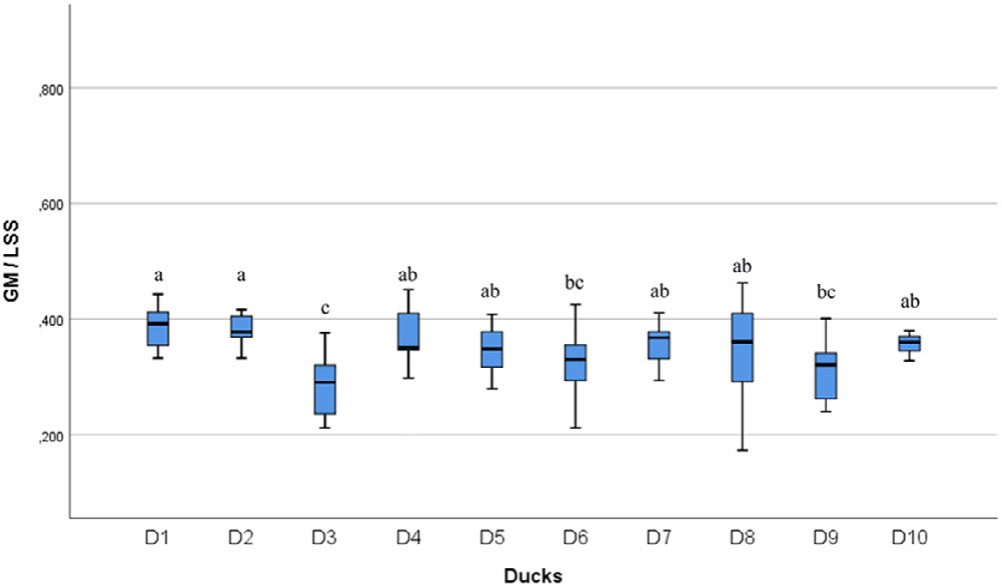
Statistical analysis for GM/LSS, ducks. The letters (a, b, c, d) indicates the differences.

**TABLE 1:** Statistical analysis of lumbosacral segments (LSS) volume of ducks (mm^3^).

		N	Mean	Standard deviation	Minimum	Maximum	Lettering	*p*
LSS	LS1	10	2.907	0.607	1.993	4.401	bcd	0.001
	LS2	10	2.864	0.698	1.995	4.606	bcd	
	LS3	10	3.449	0.960	1.977	5.172	abc	
	LS4	10	3.670	0.937	2.305	5.061	a	
	LS5	10	3.831	0.993	2.141	5.068	a	
	LS6	10	3.961	1.225	1.616	5.368	a	
	LS7	10	3.585	0.490	2.808	4.376	ab	
	LS8	10	3.728	0.703	2.522	4.835	a	
	LS9	10	2.806	0.805	1.402	4.222	cd	
	LS10	10	2.463	0.829	1.162	3.697	de	
	LS11	10	2.088	0.653	0.917	3.105	e	
	LS12	10	1.792	0.447	0.910	2.540	ef	
	LS13	10	1.282	0.386	0.800	1.700	fg	
	LS14	10	0.917	0.465	0.402	1.783	g	
	Total	140	2.810	1.198	0.402	5.368		
WM	LS1	10	1.892	0.383	1.227	2.688	abc	0.001
	LS2	10	1.847	0.440	1.238	2.786	abc	
	LS3	10	2.189	0.595	1.265	3.400	a	
	LS4	10	2.100	0.562	1.633	3.283	ab	
	LS5	10	2.118	0.441	1.277	2.868	a	
	LS6	10	2.200	0.608	0.995	3.048	a	
	LS7	10	2.021	0.258	1.641	2.431	ab	
	LS8	10	2.057	0.353	1.695	2.712	ab	
	LS9	10	1.650	0.486	0.830	2.346	bcd	
	LS10	10	1.476	0.495	0.715	2.371	cde	
	LS11	10	1.246	0.418	0.458	1.875	de	
	LS12	10	1.074	0.351	0.530	1.858	ef	
	LS13	10	0.811	0.415	0.441	1.816	fg	
	LS14	10	0.535	0.471	0.238	1.807	g	
	Total	140	1.658	0.686	0.238	3.400		
GM	LS1	10	0.875	0.184	0.618	1.141	cd	0.001
	LS2	10	0.857	0.243	0.583	1.358	cd	
	LS3	10	1.092	0.350	0.750	1.665	abc	
	LS4	10	1.210	0.337	0.611	1.680	ab	
	LS5	10	1.238	0.468	0.588	1.912	ab	
	LS6	10	1.372	0.480	0.563	1.992	a	
	LS7	10	1.255	0.348	0.642	1.861	ab	
	LS8	10	1.255	0.366	0.528	1.701	ab	
	LS9	10	0.993	0.256	0.576	1.398	bc	
	LS10	10	0.868	0.272	0515	1.427	cd	
	LS11	10	0.806	0.286	0.408	1.312	cd	
	LS12	10	0.600	0.129	0.355	0.761	de	
	LS13	10	0.498	0.108	0.345	0.648	e	
	LS14	10	0.345	0.146	0.195	0.665	e	
	Total	140	0.947	0.419	0.195	1.992		
GM/WM	LS1	10	0.471	0.107	0.312	0.649	cd	0.001
	LS2	10	0.454	0.112	0.262	0.609	d	
	LS3	10	0.498	0.107	0.295	0.614	cd	
	LS4	10	0.582	0.136	0.374	0.846	bcd	
	LS5	10	0.575	0.159	0.352	0.866	bcd	
	LS6	10	0.615	0.105	0.464	0.783	abc	
	LS7	10	0.625	0.170	0.341	0.831	abc	
	LS8	10	0.622	0.181	0.297	0.826	abc	
	LS9	10	0.622	0.115	0.316	0.697	abc	
	LS10	10	0.614	0.121	0.327	0.741	abc	
	LS11	10	0.680	0.206	0.331	1.102	ab	
	LS12	10	0.594	0.155	0321	0.775	bcd	
	LS13	10	0.686	0.176	0.339	0.986	ab	
	LS14	10	0.766	0.174	0.368	0.980	a	
	Total	140	0.600	0.163	0.262	1.102		
WM/LSS	LS1	10	0.651	0.049	0.597	0.750	*p* > 0.05 insignificant	0.217
	LS2	10	0.645	0.045	0.597	0.711		
	LS3	10	0.642	0.063	0.571	0.772		
	LS4	10	0.578	0.078	0.437	0.708		
	LS5	10	0.564	0.080	0.435	0.692		
	LS6	10	0.565	0.060	0.473	0.640		
	LS7	10	0.568	0.074	0.469	0.697		
	LS8	10	0.560	0.088	0.402	0.704		
	LS9	10	0.588	0.042	0.530	0.672		
	LS10	10	0.603	0.060	0.507	0.726		
	LS11	10	0.591	0.060	0.499	0.716		
	LS12	10	0.592	0.055	0.531	0.731		
	LS13	10	0.615	0.176	0.516	1.110		
	LS14	10	0.605	0.145	0.528	1.013		
	Total	140	0.598	0.087	0.402	1.110		
GM/LSS	LS1	10	0.300	0.048	0.234	0.396	d	0.001
	LS2	10	0.299	0.047	0.212	0.369	d	
	LS3	10	0.313	0.047	0.241	0.379	d	
	LS4	10	0.328	0.034	0.265	0.370	cd	
	LS5	10	0.316	0.060	0.178	0.377	d	
	LS6	10	0.343	0.034	0.279	0.387	abc	
	LS7	10	0.341	0.062	0.228	0.425	abc	
	LS8	10	0.325	0.064	0.209	0.403	cd	
	LS9	10	0.362	0.058	0.212	0.412	abc	
	LS10	10	0.365	0.060	0.237	0.443	abc	
	LS11	10	0.395	0.106	0.236	0.652	ab	
	LS12	10	0.349	0.084	0.173	0.451	abc	
	LS13	10	0,409	0.067	0.336	0.543	b	
	LS14	10	0.480	0.137	0.320	0.812	a	
	Total	140	0.352	0.082	0.173	0.812		

*Note*: ANOVA analysis was performed to compare group means. Differences between subgroups were determined by Duncan Multiple Comparison test. There is a statistically significant difference between groups with *p* < 0.05 and different letters. SPSS (IBM SPSS for Windows, Ver.23) statistical package program was used for statistical calculations. The letters (a, b, c, d) indicate the differences.

Abbreviations: D, duck; GM, grey matter; N, number of duck; LS, lumbosacral; LSS, lumbosacral segment; WM, white matter.

In the statistical evaluation of between animal lumbosacral volume values, the difference was insignificant in terms of whole segment and GM volume values, while the difference was significant in terms of WM, GM/WM, WM/LSS and GM/LSS values (Table 2).

**TABLE 2:** Statistical analysis between ducks for volume of WM, GM, LSS, GM/WM, GM/LSS, and WM/LSS (mm^3^).

		N	Mean	Standard deviation	Minimum	Maximum	Lettering	*p*
Segment_Duck	D1	14	2.467	1.104	0.797	4.032	*p* > 0.05 insignificant	0.134
	D2	14	2.994	1.435	0.402	5.368		
	D3	14	3.083	1.022	1.635	5.172		
	D4	14	1.920	0.834	0.897	3.318		
	D5	14	2.515	0.909	1.275	4.147		
	D6	14	3.119	1.013	1.290	4.502		
	D7	14	3.166	1.607	0.837	5.061		
	D8	14	2.885	1.120	0.797	4.301		
	D9	14	3.003	1.129	0.478	4.835		
	D10	14	2.950	1.368	0.408	5.081		
	Total	140	2.810	1.198	0.402	5.368		
WM Duck	D1	14	1.414	0.603	0.421	2.237	bc	0.005
	D2	14	1.575	0.622	0.238	2.543	bc	
	D3	14	2.191	0.566	1.347	3.400	a	
	D4	14	1.173	0.548	0.458	1.971	c	
	D5	14	1.397	0.587	0.265	2.497	bc	
	D6	14	1.853	0.521	0.705	2.262	ab	
	D7	14	1.847	0.959	0.456	3.048	ab	
	D8	14	1.691	0.641	0.431	2.421	abc	
	D9	14	1.820	0.667	0.263	2.712	ab	
	D10	14	1.621	0.668	0.241	2.581	bc	
	Total	140	1.658	0.686	0.238	3.400		
GM Duck	D1	14	0.951	0.378	0.413	1.561	*p* > 0.05 insignificant	0.112
	D2	14	1.101	0.506	0.216	1.992		
	D3	14	0.871	0.331	0.597	1.665		
	D4	14	0.650	0.255	0.361	1.158		
	D5	14	0.801	0.313	0.195	1.222		
	D6	14	1.003	0.414	0.467	1.861		
	D7	14	1.095	0.510	0.336	1.816		
	D8	14	1.037	0.463	0.355	1.701		
	D9	14	0.948	0.395	0.231	1.627		
	D10	14	1.017	0.465	0.212	1.836		
	Total	140	0.947	0.419	0.195	1.992		
GM/WM Duck	D1	14	0.708	0.131	0.541	0.986	a	0.001
	D2	14	0.708	0.137	0.433	0.907	a	
	D3	14	0.393	0.072	0.316	0.509	c	
	D4	14	0.606	0.158	0.297	0.890	ab	
	D5	14	0.595	0.108	0.352	0.741	ab	
	D6	14	0.543	0.153	0.295	0.831	b	
	D7	14	0.624	0.087	0.424	0.738	ab	
	D8	14	0.632	0.234	0.262	1.102	ab	
	D9	14	0.547	0.154	0.351	0.878	b
	D10	14	0.648	0.125	0.406	0.879	ab	
	Total	140	0.600	0.163	0.262	1.102		
WM/LSS Duck	D1	14	0.576	0.029	0.528	0.615	b	0.001
	D2	14	0.553	0.090	0.402	0.690	b	
	D3	14	0.736	0.142	0.627	1.110	a	
	D4	14	0.600	0.046	0.499	0.704	b	
	D5	14	0.572	0.036	0.507	0.639	b	
	D6	14	0.603	0.078	0.498	0.750	b	
	D7	14	0.577	0.020	0.544	0.607	b	
	D8	14	0.588	0.075	0.516	0.772	b	
	D9	14	0.606	0.070	0.507	0.708	b	
	D10	14	0.565	0.068	0.472	0.670	b	
	Total	140	0.598	0.087	0.402	1.110		
GM/LSS Duck	D1	14	0.401	0.063	0.332	0.543	a	0.003
	D2	14	0.388	0.059	0.299	0.537	a	
	D3	14	0.282	0.050	0.212	0.376	c	
	D4	14	0.358	0.073	0.209	0.451	ab	
	D5	14	0.368	0.139	0.178	0.812	ab	
	D6	14	0.320	0.057	0.212	0.425	bc	
	D7	14	0.354	0.042	0.259	0.411	ab	
	D8	14	0.369	0.113	0.173	0.652	ab	
	D9	14	0.319	0.066	0.240	0.483	bc	
	D10	14	0.360	0.055	0.272	0.519	ab	
	Total	140	0.352	0.082	0.173	0.812		

*Note*: ANOVA analysis was performed to compare group means. Differences between subgroups were determined by Duncan Multiple Comparison test. There is a statistically significant difference between groups with *p* < 0.05 and different letters. SPSS (IBM SPSS for Windows, Ver.23) statistical package program was used for statistical calculations. The letters (a, b, c, d) indicate the differences.

Abbreviations: D, duck; GM, grey matter; N, number of duck; LS, lumbosacral; LSS, lumbosacral segment; WM, white matter.

## 3 Results

In ducks, the number of segments of the lumbosacral region was determined to be 14 by dissection led by the columna vertebralis. In this study, the average volume value of the LSS was determined as 2.085 mm^3^. When all LSS were evaluated, the GM volume value was found to be 0.946 mm^3^. According to the results obtained, when the average of all segmentations was calculated, the WM volume value was determined as 1.657 mm^3^. Based on all the data, GM/WM, WM/LSS, GM/LSS ratio values in terms of volume data were also calculated and included in the results of the study. According to these calculations, the average GM/WM value for all segments was determined as 0.602. When the averages of all segments were evaluated in terms of volume ratios, the WM/LSS value was calculated as 0.597. When these values were examined in terms of GM/LSS, the average value was found to be 0.351.

In this study, the average number of points obtained in the LSS volume calculation of 10 animals was calculated as 2216. In GM volume calculation, the average of LSS in ten animals was calculated as 747. The average number of points in WM volume calculations was determined as 1320.

In this study, when all the volume values of the lumbosacral spinal segment in duck were revealed, it was determined that the volume value increased in the range of LS3 and LS8 segments. The increase in the whole volume value in these segment ranges in ducks gave rise to the idea that the enlargement called intumescentia lumbosacralis may be between these segments. In addition, it was determined that the WM volume value increased between LS3 and LS8 compared to other segments. Likewise, the increase in GM volume values between LS3 and LS8, like whole volume and WM volume values, revealed a remarkable situation. It was determined that the increase in the whole volume value between these segments was accompanied by both WM and GM volume values. The increase in the total volume, WM and GM volume values is parallel to the LS3 and LS8 segments.

## 4 Discussion

In their study, Bakıcı et al. (2019) reported that the Cavalieri's Principle is an objective, accurate and effective method in terms of volume calculations.

In a study, the spinal cord was divided into sections. At the same time, the spinal cord running along the spine is also divided into segments. After decalcification of the spine, spinal segments were exposed (Begum et al. 2010). In a study conducted in Leghorn chickens, it was reported that there is no need to separate the spinal cord segments before decalcification of the spine and that the spinal cord can be obtained as a whole after the spine is decalcified (Bolat 2011). In another study, Alizadeh et al. (2024) conducted on the skulls of Saanen goats, they washed the bones, bleached them with hydrogen peroxide, and kept them in 10% potassium hydroxide for 5 days in order to decalcify them. In this study on the LSS of ducks, the spinal cord was dissected under the leadership of the vertebral column while it was attached to the vertebral columns. In this study, it differs from both studies in that decalcification of the spine was not preferred and the segments of the spinal cord were exposed under the guidance of the spine. In a study on the skeletal system of teal (*Anas crecca*), it was revealed that the dorsal vertebrae of the synsacrum are formed by the union of all the lumbar and rump vertebrae and the first five tail vertebrae (Can et al. 2010). In this study, unlike the study of Can et al. (2010) on teal, it was determined that lumbosacral vertebrae were formed by the union of all lumbar vertebrae and sacral vertebrae in ducks. Bolat (2011) reported the presence of 14 lumbosacral vertebrae in chickens in a study the author conducted on Leghorn male and female chickens. Fourteen lumbosacral vertebrae were detected by Bolat (2011) in Leghorn chickens and 14 lumbosacral vertebrae were detected in this study on ducks, although the breeds are different, overlap in number. In a study by Uehara and Ueshima (1985) on the LSS of chickens, they reported the presence of 14 LSS. In a different study, it was stated that the lumbosacral part of pigeons consists of 21 segments (Chiasson 1964). In a study conducted in Leghorn chickens, the number of LSS was reported as 14 (Bolat 2011). In this study on the LSS of ducks, the number of segments was determined as 14. While this study on ducks overlaps with the study of Bolat (2011) and the study of Uehara and Ueshima (1985), it differs in terms of the number of segments with other studies on the LSS of poultry.

Optical dissectors were preferred in the study on the spinal cord of Leghorn chickens (Bolat 2011). In a study conducted by Selcuk (2011) on horses, although LSS volume values were calculated using the Cavalieri's Principle, the optical dissector was preferred. Although this study differs from the existing studies since physical dissector is used in terms of dissector, Cavalieri's Principle was preferred for volume value calculations. It is similar to other studies in terms of volume value calculations.

In a study in which intumescentia lumbosacralis was detected, it was reported that this part corresponds to LS1‐LS8 segments in chickens and LS1‐LS11 segments in ducks (Haziroglu et al. 2001). In a study conducted by Bolat (2011) in Leghorn chickens, it was determined that the widest part of the spinal cord was intumescentia lumbalis. In a different study by Tasbas (1978) on transversal sections of the spinal cord, it was stated that the widest part was the intumescentia lumbalis. In the study of Cakmak and Karadag (2019) on quails, it was reported that intumescentia lumbosacralis coincides with the LS1‐LS6 segments. In this study, it was determined that intumescentia lumbosacralis was found between LS3 and LS8. This result does not show agreement with the studies examined in terms of segments.

The study of Haziroglu et al. (2001) reported that the corpus gelatinosum covers the inside of the canalis centralis in the segments of LS2‐LS9 in pigeons, LS5‐LS11 in ducks and LS6‐LS10 in chickens. In a study by Bolat (2011), it was determined that the sinus rhomboidalis is filled by the corpus gelatinosum in Leghorn chickens. In this study, it was observed that the sinus rhomboidalis was filled by the corpus gelatinosum, similar to the study of Bolat (2011).

Rahmanifar et al. (2008) reported that GM was less in pars thoracalis in a study conducted in ostriches. In this study, it was determined that the GM volume in the LSS was lower than the WM volume.

In their stereological and morphometric study on LSS in New Zealand rabbits, in addition to the hematoxylin and eosin staining technique, Duman and Gurbuz (2024) also stained with Luxol Fast Blue. Our study is similar to this study in terms of the haematoxylin and eosin staining method and the use of stereological techniques. They found that WM volume was the highest in L4 for both sexes in smoke and robust rabbits. They reported that the segment with the highest GM volume is L6. In our study, it was determined that both total volume and white and GM volume increased between L3 and L8 segments. As a result, it was stated that whole volume, white and GM volume densities of the LSS in ducks can be calculated separately and that the obtained values can be revealed by stereological methods. In addition, with this study, it was observed that the sampling method should be determined separately for each segment belonging to the spinal cord in ducks. Thus, it was concluded that the physical dissector could be the right choice when determining the sampling strategy. It was determined that the volume values of the LSS in adult ducks differ in terms of all segments, WM and GM parts in 14 segments.

It has been revealed that this study can lead to stereological studies in order to clearly determine volume calculations and reference values in all anatomical, histological and neurological studies on the nervous system in poultry. In addition, we are of the opinion that this study can be of reference value in studies to be carried out in this direction, new studies can be supported by this study and it can be a light of literature for other studies. The necessity of not being limited to anatomical studies on poultry is also advocated by this study.

## Author Contributions

**Gamze Cakmak and Zafer Soyguder**: Investigation; methodology; supervision. **Gamze Cakmak**: Writing‐original draft. **Gamze Cakmak**: Conceptualization. **Gamze CAKMAK**: Data curation. **Gamze Cakmak** and **Zafer Soyguder**: Formal analysis; funding acquisition; software.

## Conflicts of Interest

The authors declare no conflicts of interest.

## Ethics Statement

This study was conducted at Van Yuzuncu Yil University Faculty of Veterinary Medicine, in accordance with the decision of the Van Yuzuncu Yil University Animal Experiments Local Ethics Committee dated 24 June 2021 and numbered 2021/06‐17.

### Peer Review

The peer review history for this article is available at https://publons.com/publon/10.1002/vms3.70289.

## Data Availability

The data that support the findings of this study are available from the corresponding author upon reasonable request.
